# Supplementary material for: A Trip Back Home: Resistance to Herbivores of Native and Non-Native Plant Populations of Datura stramonium
Source: Plants (Basel). 2024 Jan 2;13(1):131. doi: 10.3390/plants13010131 (PMC10780412; doi:10.3390/plants13010131)
Supplement: Supplementary file 1 [file plants-13-00131-s001.zip › plants-2694878-supplementary.pdf]

Supplementary Materials

A Trip Back Home: Resistance to Herbivores of Native and Non-Native Plant Populations of *Datura stramonium*

Table S1. Nested analysis of variance of traits measured in plants of *Datura stramonium* between Ranges (Mexico, Spain), Population nested with Range, and Families nested within Population, within Ranges. A.—Herbivores (*Epitrix parvula, Trichobaris soror, Sphenarium purpurascens* and *Lema daturaphila*). B.—Plant height; C.—Proportion of leaf area removed by herbivores. D.—Number of fruits per plant measured at two experimental sites (Atlixco and Teotihuacán). E.—Tropane alkaloids (atropine and scopolamine) measured only in plants at the Teotihuacán site. Statistical analyses were performed on transformed values of variables (see Results). *, $p < 0.05$.

A.- Herbivores

	Epitrix parvula					*Epitrix parvula*				
	**Atlixco**					**Teotihuacán**				
**Source of Variation**	**SS**	**df**	**MS**	***F***	**Prob>F**	**SS**	**df**	**MS**	***F***	**Prob>F**
Range	0.0023	1	0.0023	0.0053	0.9486	0.1835	1	0.1835	3.996	0.1836
Population (Range)	0.8786	2	0.4393	0.9209	0.4010	0.0918	2	0.0459	0.095	0.909
Families (Population)	31.8814	68	0.4688	0.8664	0.7430	23.6458	57	0.4850	1.098	0.3117
Error	75.7624	140	0.5411			94.4543	214	0.4413		
Total	109.0412	211				122.4200	274			

	Trichobaris soror					*Trichobaris soror*				
	**Atlixco**					**Teotihuacán**				
**Source of Variation**	**SS**	**df**	**MS**	***F***	**Prob>F**	**SS**	**df**	**MS**	***F***	**Prob>F**
Range	0.1716	1	0.7116	0.021	0.8970	4.0514	1	4.0514	0.8500	0.4530
Population (Range)	16.194	2	8.097	3.283	0.0420*	9.6430	2	4.8215	3.8704	0.0250*
Families (Population)	171.369	69	2.483	1.106	0.2990	75.625	57	1.3267	1.5587	0.0170*
Error	359.197	160	2.244			125.1241	147	0.8511		
Total	546.504	232				215.4251	207			

	Sphenarium purpurascens					*Lema daturaphila*				
	**Atlixco**					**Teotihuacán**				
**Source of Variation**	**SS**	**df**	**MS**	***F***	**Prob>F**	**SS**	**df**	**MS**	***F***	**Prob>F**
Range	9.051	1	9.051	42.33	0.0210*	0.9642	1	0.9642	25.737	0.036*
Population (Range)	0.423	2	0.211	0.369	0.6910	0.0747	2	0.0373	0.076	0.926
Families (Population)	37.279	68	0.548	0.716	0.9370	28.3894	57	0.4980	1.437	0.034*
Error	107.177	140	0.765			72.7495	210	0.3464		
Total	156.158	211				102.4629	270			

B.-Plant height

	**Atlixco**					**Teotihuacán**				
**Source of Variation**	**SS**	**df**	**MS**	***F***	**Prob>F**	**SS**	**df**	**MS**	***F***	**Prob>F**
Range	17116.90	1	17116.90	11.42	0.077	91.94	1	91.94	1.15	0.395
Population (Range)	3019.43	2	1509.72	5.07	0.008*	159.30	2	79.65	0.39	0.675
Families (Population)	21190.6	69	307.11	1.72	0.002*	11521.90	57	202.13	1.04	0.403
Error	28518.26	160	178.23			38315.31	198	193.51		
Total	74173.94	232				49963.00	258			

C.-Proportion of leaf area damaged by herbivores

	Atlixco					Teotihuacán				
**Source of Variation**	**SS**	**df**	**MS**	***F***	**Prob>F**	**SS**	**df**	**MS**	***F***	**Prob>F**
Range	0.0753	1	0.0753	4.14	0.1780	0.1695	1	0.1695	28.81	0.033*
Population (Range)	0.0365	2	0.0182	2.69	0.0740	0.0117	2	0.0058	0.23	0.78
Families (Population)	0.4775	69	0.0069	1.34	0.0655	1.3933	56	0.0248	1.21	0.17
Error	0.8226	160	0.0051			3.5084	171	0.0205		
Total	1.4351	232				5.0740	230			

D.- No. of fruits

	Atlixco					Teotihuacán				
**Source of Variation**	**SS**	**df**	**MS**	***F***	**Prob>F**	**SS**	**df**	**MS**	***F***	**Prob>F**
Range	0.0176	1	0.0176	0.01	0.9203	2.0842	1	2.0842	1.94	0.297
Population (Range)	2.7839	2	1.3919	3.84	0.0255*	2.1408	2	1.0704	2.15	0.123
Families (Population)	25.0522	69	0.3630	1.02	0.4325	28.8044	57	0.5053	1.24	0.139
Error	56.4242	160	0.3526			75.4348	186	0.4055		
Total	84.6675	232				109.1682	246			

E.- Alkaloids, Teotihuacán

	Atropine					Scopolamine				
**Source of Variation**	**SS**	**df**	**MS**	***F***	**Prob>F**	**SS**	**df**	**MS**	***F***	**Prob>F**
Range	0.5225	1	0.5225	0.72	0.483	0.5755	1	0.5755	0.38	0.596
Population (Range)	1.4642	2	0.7321	2.65	0.077	3.0135	2	1.5067	3.88	0.025*
Families (Population)	13.5551	50	0.2711	0.87	0.693	20.1718	50	0.4034	1.45	0.052
Error	35.8149	116	0.3087			32.2515	116	0.2780		
Total	52.7497	169				55.7017	169			

Table S2. Geographic location and environmental characteristics of the (A) Experimental common gardens and (B) sampled populations of *Datura stramonium* of each range (Spain or Mexico). (C) Number of families studied per population in each experimental site. (D) Average number (± S.E.) of adults, egg clusters and larvae of the insect *Lema daturaphila* on plants of *D. stramonium* of native and non-native populations.

(A).- Experimental common gardens

Locality	State or Region	Geographic coordinates	Altitude m a. s. l.	Mean Annual Precipitation (mm)	Mean Annual temperature (° C)
Atlixco	State of Puebla	18°54′45″N 98°25′40″W	1834	1248	18.6
Teotihuacán	State of Mexico	19°41′33″N 98°50′38″W	2280	823	15.3

(B).- Locality of sampled populations

Locality	State or Region	Geographic coordinates	Altitude m a. s. l.	Mean Annual Precipitation (mm)	Mean Annual temperature (° C)
Teotihuacán	State of Mexico	19°41′33″N 98°50′38″W	2280	823	15.3
Ticumán	State of Morelos	18°45′40″N 99°07′09″W	972	1150	21.3
Valdeflores	Sevilla, Spain	37° 843′ 2.23″N 06°818′50.44″W	287	598.2	17.0
Zubia	Granada, Spain	37°07′14″N 03°35′06″W	153	453	15.3

(C). -Number of families studied per population in each experimental site.

	Experimental site	
Population	Atlixco	Teotihuacán
Teotihuacán	19	15
Ticumán	17	15
Valdeflores	21	15
Zubia	16	16

(D).- Average values (± S.E.) of the number of adults, egg clusters and larvae of the insect *Lema daturaphila* on plants of *Datura stramonium* of native and non-native populations.

Population	N	# Adults	# Egg clusters	# Larvae
Teotihuacán	74	0.939 ± 0.198	4.530 ± 0.541	1.522 ± 0.200
Ticumán	72	0.985 ± 0.235	4.493 ± 0.680	1.838 ± 0.327
Valdeflores	68	0.667 ± 0.207	3.862 ± 0.528	1.031 ± 0.190
Zubia	79	0.694 ± 0.198	4.932 ± 0.627	1.486 ± 0.245
